# Continuous Blood Pressure Estimation Using Exclusively Photopletysmography by LSTM-Based Signal-to-Signal Translation

**DOI:** 10.3390/s21092952

**Published:** 2021-04-23

**Authors:** Latifa Nabila Harfiya, Ching-Chun Chang, Yung-Hui Li

**Affiliations:** 1 Department of Computer Science and Information Engineering, National Central University, Taoyuan 32001, Taiwan; latifanaharfiya@g.ncu.edu.tw; 2 Department of Computer Science, University of Warwick, Coventry CV4 7AL, UK; ching-chun.chang@warwickgrad.net; 3 AI Research Center, Hon Hai Research Institute, Taipei 114699, Taiwan

**Keywords:** blood pressure estimation, photopletysmography, deep learning, LSTM, autoencoder, signal-to-signal translation

## Abstract

Monitoring continuous BP signal is an important issue, because blood pressure (BP) varies over days, minutes, or even seconds for short-term cases. Most of photoplethysmography (PPG)-based BP estimation methods are susceptible to noise and only provides systolic blood pressure (SBP) and diastolic blood pressure (DBP) prediction. Here, instead of estimating a discrete value, we focus on different perspectives to estimate the whole waveform of BP. We propose a novel deep learning model to learn how to perform signal-to-signal translation from PPG to arterial blood pressure (ABP). Furthermore, using a raw PPG signal only as the input, the output of the proposed model is a continuous ABP signal. Based on the translated ABP signal, we extract the SBP and DBP values accordingly to ease the comparative evaluation. Our prediction results achieve average absolute error under 5 mmHg, with 70% confidence for SBP and 95% confidence for DBP without complex feature engineering. These results fulfill the standard from Association for the Advancement of Medical Instrumentation (AAMI) and the British Hypertension Society (BHS) with grade A. From the results, we believe that our model is applicable and potentially boosts the accuracy of an effective signal-to-signal continuous blood pressure estimation.

## 1. Introduction

As a result of the reciprocal relationship between environmental, physical, and emotional factors, blood pressure (BP) will always be a fluctuating hemodynamic phenomenon. Take white-coat hypertension case as an example, a normotensive subject often diagnosed as hypertensive over clinic BP measurement. The measurement hence becomes normal again outside the medical environment. This indicates that the true subject’s BP is influenced by the individual emotion that might be due to high anxiety. Therefore, instead of monitoring BP intermittently, performing continuous BP monitoring is crucial to assess the true subject’s BP amidst BP variability (BPV) [1,2].

Photoplethysmography (PPG) is widely used as a tool to detect the blood volume changes in the microvascular. Due to its simplicity and noninvasiveness, PPG sensor has been widely used in wearable devices, mostly for the fitness tracking feature [3]. The morphological analysis of PPG has been applied in vascular assessment, such as evaluating heart rate, vascular aging, and blood oxygen saturation [4,5]. While the PPG waveform reflects the amount of blood in a measuring site, the amount of blood itself is closely related to blood flow, which is formed from the pressure. Thus, PPG can be depicted as a potential alternate to obtain the arterial blood pressure (ABP) for continuous BP monitoring.

**Figure 1:**
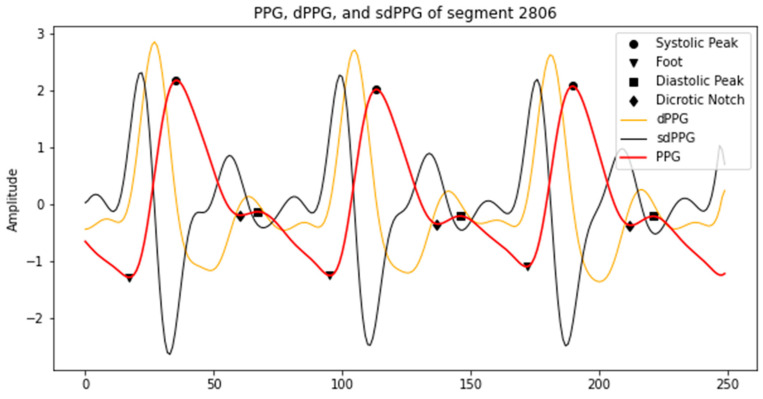
An example of PPG, dPPG and sdPPG, as well as feature points in them (peak, foot and dicrotic notch).

The general PPG waveform mainly consists of four distinctive features, namely foot, systolic peak, dicrotic notch, and diastolic peak, as shown in Figure 1. The PPG waveform is quite simple and straightforward, but sometimes is not informative. Frequently, the subject’s age affects the distinctiveness of the features, such as dicrotic notch, which is usually hard to detect in older subjects [5,6]. To conduct a better morphological analysis of PPG, most studies extend to the derivative-based analysis. The first derivation of PPG (dPPG) interprets the velocity of blood volume change and the index of blood flow. Consequently, the acceleration of the blood volume change can be captured by the second derivation (sdPPG) [4].

Earlier findings [7,8] disclose the relationship between the second derivative of PPG and blood pressure. Based on the studies, the change of PPG’s acceleration is highly related to vessel condition, such as the vessel’s radius, thickness, and stiffness. Moreover, based on [9], BP knowingly correlates with the pulse wave velocity (PWV), which comprises same information from the vessel, represented as:

(1) $$PWV=\sqrt{\frac{E\cdot h}{2\cdot r\cdot \rho }},$$

where the vessel’s radius and thickness are denoted as r and h, respectively. The vessel’s stiffness has a nonlinear relationship with the elastic modulus of the vessel wall, denoted as E, and lastly, ρ denotes the density of blood in the vessel. Therefore, we can conclude that the PPG’s derivatives can be utilized to preserve the informative features in the PPG waveform.

Prior studies focusing on the BP estimation task can be observed dealing with two kind of approaches, using either PPG signal only or PPG signal along with other signals. In [9,10,11], feature-based methods from PPG and electrocardiogram (ECG) signals are carried out. Using the same source from [12] for the ECG, PPG, and ABP signals as the training and testing set, these studies extract informative features based on the physiological parameters and the time-related indicators. The features serve as an input to machine learning and deep learning model to predict the SBP and DBP. Despite the good result in DBP prediction, these studies remain with high error in SBP prediction.

Recent studies [11,13] try to compare the performance of BP assessment between machine learning models that use the combination features from ECG and PPG signals and models that use features from PPG only. It is reported from both studies that using combination features from ECG and PPG signals results in a comparatively better performance. However, the reported results could not surpass the other studies [6,7,14,15,16] that produce remarkable results using PPG signal only. Despite the simplicity of PPG waveform, numerous features can be extracted from the time and frequency domain of an appropriate PPG signal. Estimating BP with one signal only is also more convenient when considering how the computational and hardware cost can be reduced. Hence, many studies try to focus on the task by utilizing PPG signal only.

Our prior work [14] selects 59 out of 65 total features extracted from PPG signal as the input of the deep neural network (DNN) model to predict SBP and DBP. The resulting errors are exceptionally low from both SBP and DBP prediction. This result is outperformed by [16], which selects 7 out of 22 features only as the input of recurrent neural network (RNN) models, namely long short-term memory (LSTM) and gated recurrent units (GRU). This may demonstrate the aptitude of RNN in solving sequence problem with less features as the input. The smaller number of features may also save the computational cost of the model.

Notwithstanding the effectiveness of using PPG signal for BP estimation, motion artefacts are often found diminishing the signal quality which caused feature extraction failure. Several studies [17,18,19] have attempted to use the raw signal and discard feature engineering. The raw signal is preprocessed and segmented for some defined time length. Subsequently, the signal segments are fed as inputs of the model. Several machine learning regressor models, namely decision tree, support vector regression, adaptive boosting, and random forest are being compared in [18] to predict SBP, DBP, and mean arterial blood pressure (MAP) values. Convolutional layers and recurrent layer of GRU are applied to build an end-to-end neural network model in [19] to estimate SBP and DBP values. Again, using the recurrent layer helps the performance boosts. The notable performance, however, lacks generality, since the data that they used are the least frequent compared to the other works. Lastly, a deep learning model comprised of fully convolutional neural networks is developed by [17], not only to estimate the SBP, DBP, and MAP, but also to predict the waveshape of the ABP signal. To the best of our knowledge, unlike the already mentioned works, this model is the only model that uses a signal-to-signal translation approach in the field. This model, however, consists of two separate networks, which is computationally expensive.

In this study, we develop a LSTM-based autoencoder to translate ABP signal from raw PPG signal along with its derivations, and extract the SBP and DBP values from it. The contribution of this study includes:

1. A unimodal which consists of LSTM and autoencoder as the signal-to-signal translator to estimate ABP signal using raw PPG signal only.
2. Our model has the strong learning ability to estimate the ABP signal. The input of the proposed model is raw PPG signal along with its derivatives, instead of the hand-crafted feature of the PPG. There is no feature engineering needed for the proposed model.
3. Instead of estimating discrete value (such as SBP and DBP), our model is able to estimate the whole waveshape of the ABP signal, which provides more holistic information of ABP if applied in the healthcare domain for patients with serious cardiovascular disease (CVD).

The rest of this paper is organized into four sections. Section 2 explains the database, preprocessing, and the model composition, along with the experimental setup. The experimental results are presented in Section 3. Section 4 summarizes and discusses the result, while Section 5 concludes this study.

## 2. Materials and Methods

In this study, PPG signals are acquired from Physionet’s MIMIC (Multiparameter Intelligent Monitoring in Intensive Care) II online database [12]. The corresponding ABP signals are also provided, which we use as a reference during the experiment. The sampling frequency of both signals are 125 Hz, with the length of signals varying across 12,000 subjects. It is important to note that this database obtains data from ICU, which likely contain abnormal BPV, due to influence from drugs [10]. We prepare the PPG signals through some preprocessing, and left with data from 5289 subjects to further be used in the experiments. The preprocessing steps and model building are summarized below.

### 2.1. Data Preprocessing

#### 2.1.1. Denoising

Prior to any process to be carried out, PPG signals are being filtered using third order Butterworth bandpass filter to remove both high and low-frequency noise. This filter is highly used to produce PPG signals filtration in prior works such as [4,20,21]. In this study, we specify the range of the passband from 0.5 to 8 Hz.

#### 2.1.2. Z-Score Normalization

In this step, we modify the raw data (PPG signal) so that it fits to a normal distribution. Suppose the mean of a signal is computed as μ and standard deviation as σ; then, we transform our data so that the transformed data has zero mean and standard deviation of one by:

(2) $$x^{\prime }=\frac{x-\mu }{\sigma },$$

where x is the original PPG signal and x′ is the normalized PPG signal. Here, the normalization parameters are calculated for each signal separately. Moreover, we take the average of each normalization parameter from all the data in the training stage and use it for all signals in the testing stage.

#### 2.1.3. Signal Alignment

In order to handle one known issue in the database which is waveform misalignment, we measure a cross-correlation function g(∆t) to calculate the precise phase lag between the PPG and the reference ABP signal [15]. The cross-correlation function is presented as follows:

(3) $$g\left(∆t\right)=\sum^{\;}ABP\left[t\right]PPG\left[t+\Delta t\right]$$

**Figure 2:**
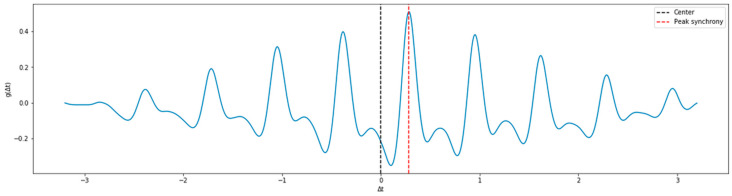
Time offset between PPG and ABP signal of example record.

**Figure 3:**
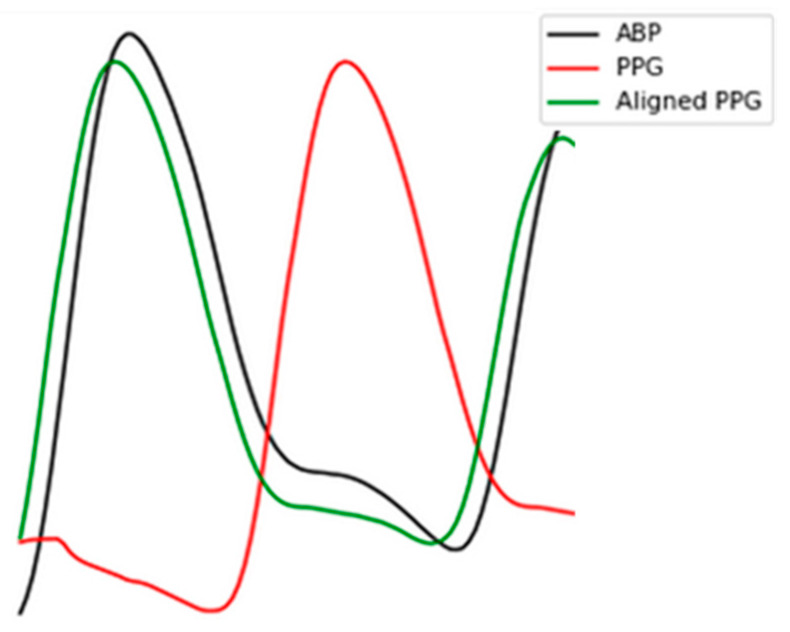
The result of signal alignment. The black and red curve denote the original ABP and PPG signal, respectively. The green curve denotes the shifted PPG signal aligned with ABP.

Here, signals are aligned for every segment with g(Δt) represents the result of cross-correlation function, and Δt is the time offset or the phase lag between ABP and PPG. The most accurate Δt corresponds to the biggest g(Δt). Δt is generally bigger than 0 and smaller than the duration of a single pulse. In Figure 2, we present an example of the function g(Δt). If the peak of cross-correlation is at the center, then it indicates that the two signals are the most synchronized. However, in Figure 2 we can see that there is a phase lag of 0.28 s in the example record, which indicates that indeed one of the signals (in our case is the ABP) leads the other signal. In such a case, we shift the PPG signal for 0.28 s in a direction closer to 0, as demonstrated in Figure 3.

#### 2.1.4. First and Second Derivative of PPG Signal Extraction

The general PPG waveform is quite simple and straightforward, yet not always informative [5]. Considering that the PPG represents how much amount the blood volume changes, taking its first and second derivatives can significantly help in evaluating the informative features in PPG waveform, such as the velocity and the accelerations of blood volume changes [4,14]. We take the derivatives of PPG waveform and stack it along to be the input of our model. Let x as a PPG data point at time i with h step size which is evenly spaced; we calculate the derivatives by the equation as follows:

(4) $$f^{\prime }\left(x_{i}\right)≅\frac{f\left(x_{i+1}\right)-(x_{i-1})}{2h}+Ο(h^{2})$$

#### 2.1.5. Elimination of Inappropriate Signals

**Figure 4:**
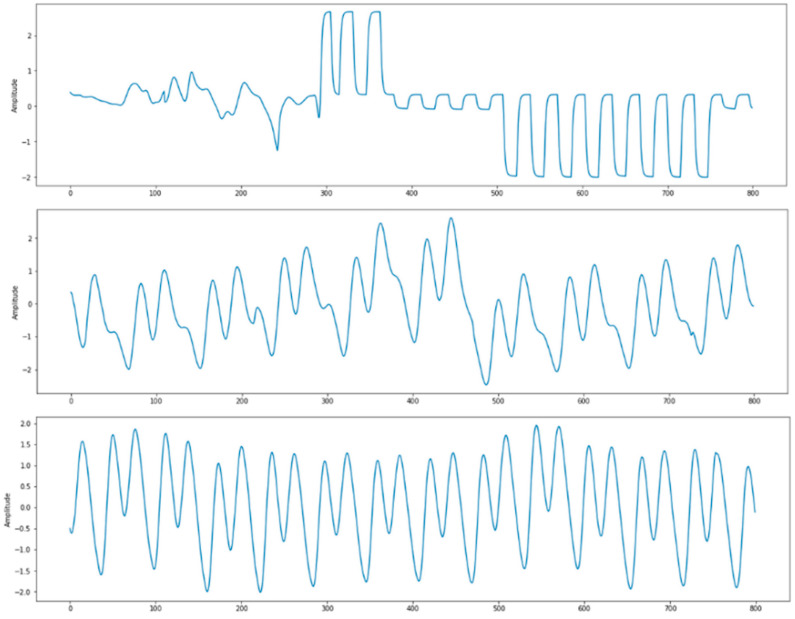
Problematic PPG signals with undefined systolic peak.

For training, we eliminate signals that do not meet our requirements, such as:

- Signal with systolic blood pressure (SBP) more than 180 mmHg or less than 80 mmHg. SBP can be calculated following this equation:
  (5) SBP=max(ABP)
- Signal with diastolic blood pressure (DBP) more than 130 mmHg or less than 60 mmHg. DBP can be calculated following this equation:
  (6) DBP=min(ABP)
- Signal with average Pearson’s correlation coefficient less than 0.8. After each beat of the signal is aligned, we compute the correlation coefficient r to determine how similar PPG and the reference ABP signal in terms of morphology by the equation as follows [3]:
  (7) $$r=\frac{n\;\sum^{\;}AP-\sum^{\;}A\sum^{\;}P}{\sqrt{n\;\sum^{\;}A^{2}-{\left(\sum^{\;}A\right)}^{2}}-\sqrt{n\;\sum^{\;}P^{2}-{\left(\sum^{\;}P\right)}^{2}}}$$
- Signal with undefined PPG systolic peak. We use heartpy toolkit [22] for the automatic detection of PPG systolic peak. The cases of undetected systolic peak mostly happened to PPG signals that have irregular waveform, which might be influenced by sensor position change or movements. A few examples are shown in Figure 4.

**Figure 5:**
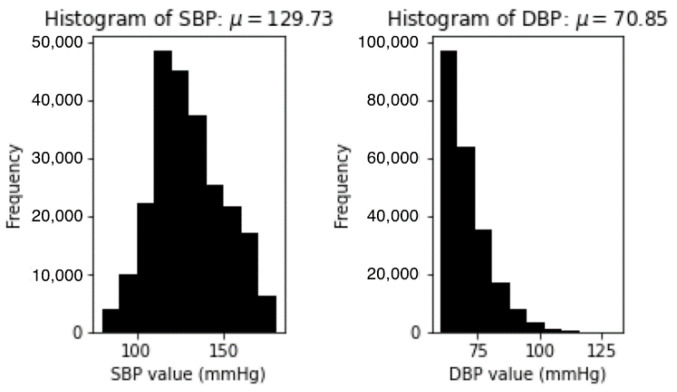
Distribution of SBP and DBP values in the final set.

The final set consists of about 250,000 segments of signal extracted from all the subjects randomly selected in the database. Figure 5 demonstrates the statistical information about the distribution of the SBP and DBP in the final set.

### 2.2. Model Building

#### 2.2.1. LSTM

**Figure 6:**
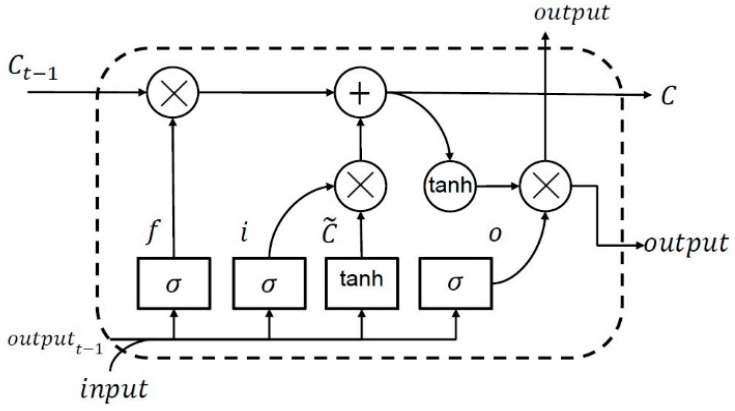
Flowchart of the LSTM module.

Sequence data, namely time series and natural language, hold a long-term dependency which requires that important information from any state is preserved. RNN are supposed to overcome the shortcoming that traditional neural network has in handling problems related to these sequential data [23]. However, as the sequence gets longer, the performance of RNN is deteriorating in some way. LSTM is one kind of RNN that is tailored to address this issue [24]. As depicted in Figure 6, the LSTM module, or so-called memory cell, comprises a unique composition that allows information to persist and pass on over time. The form of LSTM memory cell is designed to have a forget gate (f), an input gate (i), an output gate (o), and a cell state (c).

The cell state allows information to surge along it, while the gates are in charge of regulating the interaction between the memory units to determine whether the information needs to be added or removed. In particular, following the flow of LSTM memory cell, the forget gate can decide to detach information from the cell state. It looks at the output of previous memory cell at time t−1 and the current input at time t to output a number between 0 to 1 with a sigmoid (σ) function. Next, the input gate determines whether the new input is going to be stored in the cell state or not. Beside utilizing a sigmoid (σ) function that decides which values are going to be updated, it also uses hyper tangent (tanh) function to output a vector of candidate values ($\tilde{C}$) that is going to be stored in the cell state. The result from forget gate is multiplied with the cell state from previous memory cell and added to the multiplication of input gate and the candidate value accordingly to update the cell state. Lastly, the output gate filters the information from cell state as the output, by first using the sigmoid function and multiplying it by the hyper tangent of the updated cell to push the output values to be in the range of [−1, 1].

#### 2.2.2. Autoencoder

**Figure 7:**
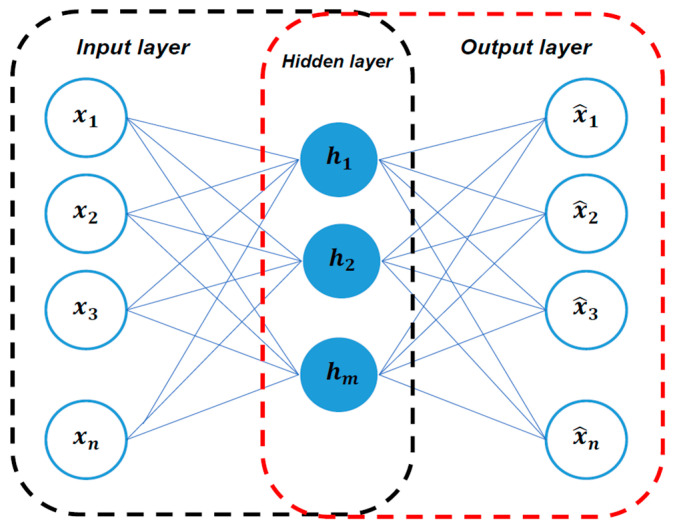
Autoencoder architecture. The encoding phase is represented in the black-line box and the decoding phase is represented in the red-line box.

The autoencoder is a kind of artificial neural network (ANN) that learns in an unsupervised technique. Often, we find autoencoder is utilized as generative model or to solve machine translation and image segmentation tasks [25]. The autoencoder has an essential composition which comprises three consecutive layers, as shown in Figure 7, to output exactly the same data as the input that it is being fed to [26]. The concept of autoencoder is unusually straightforward. The training procedure can be dissembled into two phases, encoding and decoding. In the encoding phase, the encoder first receives the input data in input layer, learns the compressed representation, and map it into the hidden layer. Inversely, decoder reconstructs the input data from the compressed representation in hidden layer during the decoding phase. To suppress the disparity between the original input and the output, the autoencoder measures the reconstruction error by computing the difference between the original input and the output. In this sense, focusing on reducing the reconstruction error that has the same input and output takes autoencoder to self-supervised learning, since the data we process provides the supervision.

#### 2.2.3. LSTM-Based Autoencoder

**Figure 8:**
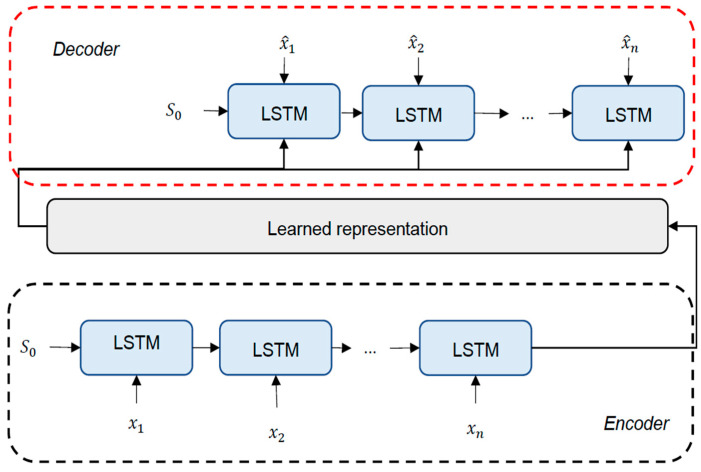
The proposed LSTM-Autoencoder model architecture.

Here, we propose a new approach for BP estimation model derived from an improved autoencoder model. Since PPG and ABP are both one-dimensional time-series signals, we believe that RNN is a more suitable tool for modelling such data. Hence, instead of using the original feed-forward neural network as the base of the autoencoder, we adopt LSTM and enable it to learn the features from the sequence in BP estimation task. The proposed model can be seen in Figure 8.

#### 2.2.4. Transfer Learning

**Figure 9:**
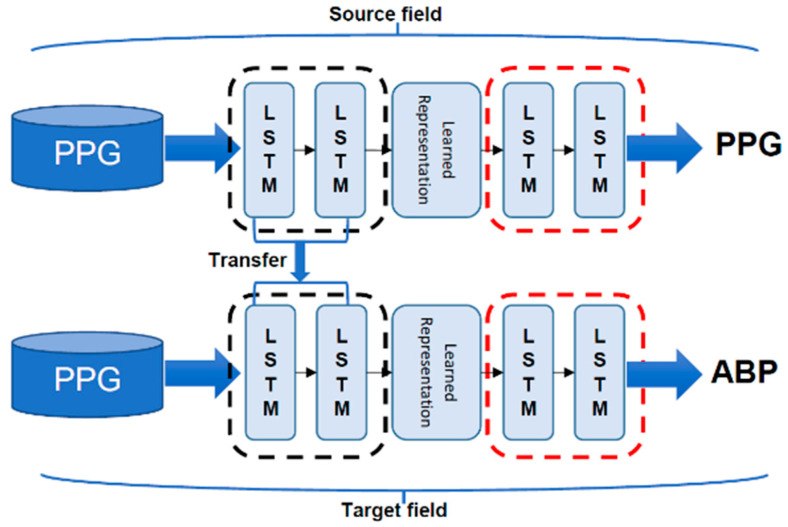
Our model training flow with transfer learning.

In machine learning, we study how to utilize knowledge from a source field and transfer it to the target field by doing transfer learning. A neural network is similar to the processing mechanism of the human brain, which comprehends an iterative and abstraction process. Following the assumption that the forepart of a network can be considered as a feature extractor while the extracted features are versatile, we can do transfer learning by reusing the partial network that trained in the source field and transfer it to be a part of a network that will be in the target domain [25]. In this study, we first train our autoencoder to reconstruct the PPG waveform input. We freeze the encoding part and only let the next part be trained for constructing the ABP waveform afterwards. Taking this application can help our network to learn the intermediate waveform representations explicitly. The training flow of our model is illustrated in Figure 9.

#### 2.2.5. Experimental Setup

In our proposed model, there are two layers of LSTM, with 128 hidden nodes for each phase. We apply dropout layer in the end of decoding phase with a rate of 0.2 to prevent overfitting. Our model’s learning rate is set to 0.0025 and uses Adam optimizer for the training process. The maximal epochs for the training are set to 50 for both source field and target field. We built our model in Python using Keras with Tensorflow 2.2 as the backend. Using four GPU machines (NVIDIA Titan X from Taipei, Taiwan), it takes up to 9 h to train both the PPG to PPG translation model and the PPG to ABP translation model.

## 3. Results

In this section, the BP estimation performances are compared with the past studies by the mean absolute error (MAE), as defined in Equation (8):

(8) $$MAE=\frac{1}{N}\sum_{i=1}^{N}\left|e_{i}\right|,$$

and the root mean square error (RMSE), as defined in Equation (9):

(9) $$RMSE=\sqrt{\frac{1}{N}\sum_{i=1}^{N}e_{i}{}^{2}},$$

where e is the error or difference between the observed and the prediction BP value in mmHg. We conduct a fair experiment by completely disjoining our data partition with ratios of 70%, 10%, 20%, for the training, validation, and testing process, respectively. The testing results are presented in Table 1.

Here, we select previous studies that also use PPG and ABP signals from the same dataset in this paper to compare. Moreover, despite using the same dataset, some methods are based on feature engineering, while others are not. Liu et al. [7] introduce 14 features from the second derivative of PPG signal and add up 21 features from prior work [27] as the input for support vector machine regressor (SVR). The reported result shows better performance than using 21 features only based on the MAE and RMSE. Wang et al. [19] demonstrates an exceptional result using free handcrafted feature engineering method and only relies on the convolutional layer as the feature extractor. However, this study uses the least number of subjects, which cannot ensure the robustness in term of generalizability. ElHajj et al. [16] have the remarkable result with the least error of DBP prediction using 7 features as the input of the GRU model. Despite that, the number of subjects is also small compared to ours. Our latest work [14] presents 65 features and select 59 features as the input of four layers deep neural network (DNN) model to achieve the smallest SBP prediction error with the largest number of subjects. Ibtehaz et al. [17] have the most similar objective to our study, which is trying to translate PPG signals to ABP signals. In their study, the model is mainly consisting of an approximation network and a refinement network. The two networks use two different image segmentation models that have been modified to handle one-dimensional data instead of two.

The results of the comparison between our proposed network and the British Hypertension Society (BHS) [28] standard are presented in Table 2. This standard grades BP measurement system, based on the cumulative error, to be less than their three different thresholds (5 mmHg, 10 mmHg, and 15 mmHg) [28]. In accordance with it, the BP estimation from proposed LSTM-based autoencoder is apparent to be consistent with the grade A for both a diastolic and systolic one.

Lastly, the evaluation of our proposed model based on Association for the Advancement of Medical Instrumentation (AAMI) [29] standard is presented in Table 3. To satisfy this standard, the test has to be conducted on more than 85 subjects with average prediction result error and standard deviation error (STD) below 5 mmHg and 8 mmHg, respectively. With respect to the mentioned criterion, our model satisfies all the standard with MAE and STD of 4.05 and 4.42 for SBP prediction also MAE and STD of 4.60 and 3.47 for DBP prediction, respectively.

## 4. Discussion

In this study, a self-supervised deep learning model for continuous BP estimation was proposed using PPG exclusively as the input. Due to its versatility and non-invasiveness, PPG becomes a widely preferred technology for obtaining information about the cardiovascular and respiration system. Features extracted from PPG have been proven to exhibit intrinsic physiological appliance, which is ideal for BP estimation [30]. Nevertheless, there had been some disputes about how susceptible PPG is influenced under various scenarios, which makes feature extraction impractical. Different from the feature-based BP estimation models, we do not perform feature engineering, in consideration of envisaging PPG in the wild, which might suffer physiological information loss.

### 4.1. Basis for PPG and ABP Signal Coherence

**Figure 10:**
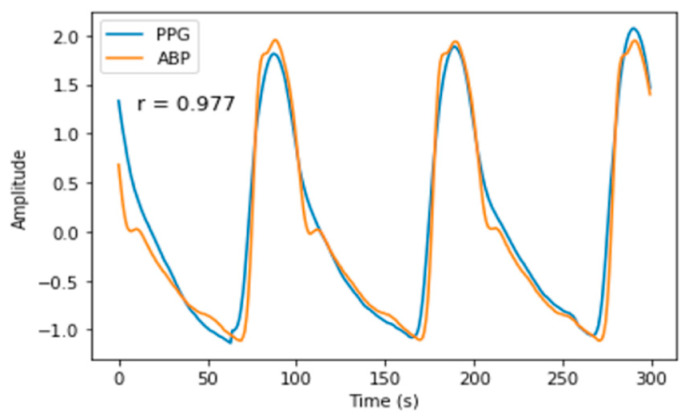
Example of in-phase analysis. The orange curve denotes the ABP signal, while the blue curve denotes the PPG signal.

We carry out an in-phase analysis where PPG and ABP have been aligned in the preprocessing part. Using the Pearson’s correlation coefficient r, we can see the similarity between the two signals in terms of morphology. From the overall 12,000 subjects, first we obtained 4,028,466 segments and selected the segment with r more than 0.8. Here, we attained 3,841,600, which means that 95% of the input signal is highly correlated with the reference signal. The average morphology correlation between PPG and ABP signal is 0.84 and Figure 10 shows one example of the segment that had quite a high positive similarity, with morphology correlation between the signals of 0.977. This finding reinforces our study in performing BP estimation without having to do complex feature engineering.

### 4.2. Model Performance

**Figure 11:**
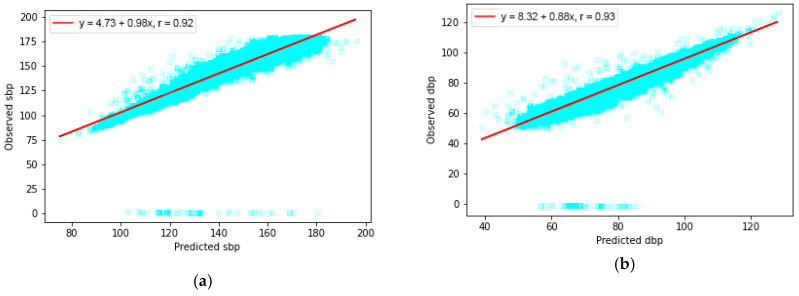
Pearson’s correlation analysis between (**a**) predicted and observed SBP, (**b**) predicted and observed DBP.

LSTM has a complex cell architecture which carries more weight than the conventional feed-forward neural network. Such a characteristic enables our model to analyze and ultimately capture more information from the sequence signal. Unlike the feed-forward neural network, LSTM also handles varying length series straightforwardly, which is able to deal with our task efficiently. In our case, we can view our goal as a signal-to-signal translation, since an arbitrarily long PPG input sequence is mapped to ABP sequence as output, with the same length as the input. From Figure 11, the results show that the Pearson’s correlation’s r is equal to 0.92 between observed and predicted SBP, and that r is equal to 0.93 between the observed and predicted DBP, indicating a high correlation between our estimation result with the ground truth.

**Figure 12:**
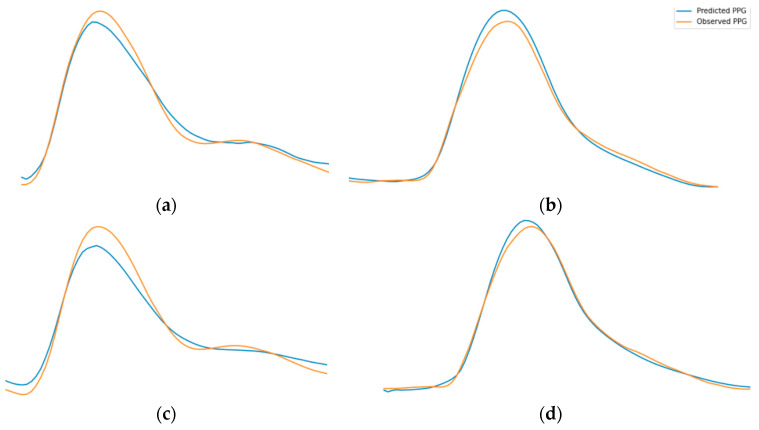
Examples of PPG prediction result from PPG to PPG reconstruction model. The subfigures indicate the observed and predicted randomly selected PPG of (**a**) segment id 12, (**b**) segment id 569, (**c**) segment id 7111, (**d**) segment id 8019.

**Figure 13:**
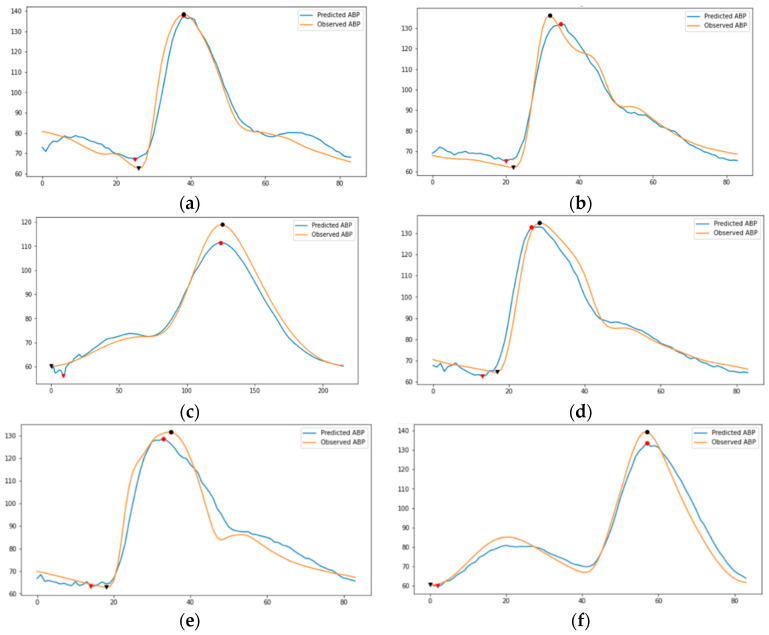
Examples of ABP prediction results from proposed model. The circle marks indicate SBP and the triangle marks indicate DBP. The subfigures indicate the observed and predicted randomly selected ABP of (**a**) segment id 4, (**b**) segment id 11, (**c**) segment id 400, (**d**) segment id 2662, (**e**) segment id 9000, (**f**) segment id 132,290.

In autoencoder, first, we need the encoder to understand the morphology of the PPG signal. It further creates the learned representation of what the PPG signals looks like. Consequently, we need the decoder to be capable of reconstructing the representation of PPG signal back into the PPG signal. By doing this iteratively through training, the encoder becomes conversant in creating the learned representation of PPG signal. Figure 12 shows the examples of PPG to PPG reconstruction model training results. Since the encoder part has been trained correctly, the decoder’s task is changed to convert the representation of PPG signal into an ABP signal. Thus, we do transfer learning for the encoder part to be used in the proposed model for estimating the ABP signal. Figure 13 demonstrates our ABP sequence prediction result using the transfer learning method, which has high resemblance to the observed sequence obtained from the source dataset. In this sense, it is certain that an LSTM-based autoencoder can perceive the information in the PPG signal and translate it to the corresponding ABP signal.

**Figure 14:**
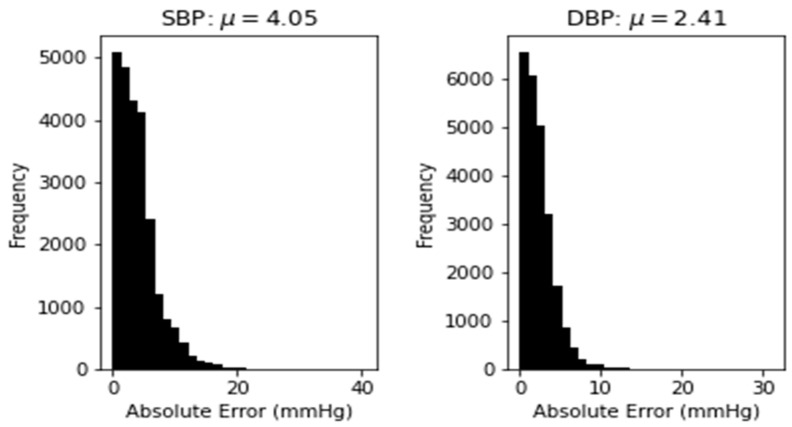
Distribution of absolute error for SBP prediction (**left**) and DBP prediction (**right**).

In our experiment, the number of subjects we use is much bigger than those used in some prior studies mentioned in Table 1 as a comparison. Although we have slightly higher error, our approach can estimate the whole waveform of an ABP signal period. The estimated waveform contains more information that can be used as reference for doctors to make a medical diagnosis. Compared to [17], that has the closest objective to the proposed method, our prediction has lower error on both SBP and DBP. With respect to it, our model shows acceptable robustness and accuracy over a fairly large number of subjects being used. In addition to that, we also emphasize the data quality, as we rely on clean PPG and ABP signal with a high correlation for our model training. Figure 14 demonstrates the distribution of absolute error across 250,000 records of SBP and DBP. It can be seen that the absolute error values follow the half normal distribution around zero. Nevertheless, the elimination process of abnormal (hypertensive and hypotensive) SBP and DBP values relatively reduce the dataset up to 90%. Hence, our limitation lies on the average number of records in each subject, which is relatively small despite the large number of subjects that we use. A training model with such data can cause the proposed approach to not be valid in some unusual cases. Given this, a wider range of SBP and DBP values might be conducted for future works with more intricate computational and analysis to support the model’s performance.

## 5. Conclusions

An LSTM-based autoencoder model to translate PPG signals toward ABP signals for blood pressure estimation has been developed. Our model omits the impractical complex feature engineering approach, which sometimes cannot be conducted due to the various scenario during acquisition that might influence the PPG signal’s quality. The model provides a fairly accurate and promising result over a very large number of subjects being examined. According to the BHS standard, our proposed model achieves grade A for both SBP and DBP estimation. We also fulfill the requirement of the AAMI standard consistently for both standards. In future works, we would like to explore our model robustness with a wider range of SBP and DBP values, especially by including abnormal BP cases.

## Figures and Tables

**Table 1:** Performance comparison (in terms of MAE and RMSE) between different methods.

Method	Dataset	SBP (mmHg)		DBP (mmHg)	
		MAE	RMSE	MAE	RMSE
[7]	910 subjects	8.54	10.9	4.34	5.8
[19]	90 subjects	3.95	-	2.14	-
[16]	500 subjects	3.25	-	1.43	-
[14]	9000 subjects	3.21	4.63	2.23	3.21
[17]	942 subjects	5.73	-	3.45	-
Proposed model	5289 subjects	4.05	5.25	2.41	3.17

**Table 2:** Performance comparison with the BHS standard.

Cumulative Error		≤5 mmHg	≤10 mmHg	≤15 mmHg
Our result	SBP	70.6%	94.1%	98.6%
	DBP	91.1%	99.1%	99.8%
BHS	Grade A	60%	85%	95%
	Grade B	50%	75%	90%
	Grade C	40%	65%	85%

**Table 3:** Performance comparison with the AAMI standard.

		MAE (mmHg)	STD (mmHg)	# Subjects
Our result	SBP	4.05	4.60	5289
	DBP	2.41	3.11	5289
AAMI		<5	<8	>85

## Data Availability

In this study, we use a publicly available dataset, MIMIC II, that can be found here: https://archive.ics.uci.edu/ml/datasets/Cuff-Less+Blood+Pressure+Estimation (accessed on 22 April 2021).
